# Identification of reliable biomarkers of human papillomavirus 16 methylation in cervical lesions based on integration status using high-resolution melting analysis

**DOI:** 10.1186/s13148-018-0445-8

**Published:** 2018-01-23

**Authors:** Lu Liu, Chunmei Ying, Zhen Zhao, Long Sui, Xinyan Zhang, Chunyan Qian, Qing Wang, Limei Chen, Qisang Guo, Jiangnan Wu

**Affiliations:** 1 Department of Clinical Laboratory, The Obstetrics and Gynecology Hospital of Fudan University, Fangxie Road No. 419, Huangpu District, Shanghai, 200001 China; 2 Department of Clinical Laboratory, Minhang District Central Hospital, Shanghai, China; 3 Medical Center of Diagnosis and Treatment for Cervical Diseases, The Obstetrics and Gynecology Hospital of Fudan University, Fangxie Road No. 419, Huangpu District, Shanghai, 200001 China; 4 The Research Institute of Obstetrics and Gynecology, The Obstetrics and Gynecology Hospital of Fudan University, Shanghai, China; 5 Yuhang Branch, Second Affiliated Hospital of Zhejiang University, Hangzhou, China; 6 Department of Clinical Statistics, The Obstetrics and Gynecology Hospital of Fudan University, Shanghai, China

**Keywords:** Squamous cervical cancer, Integration, High-resolution melting analysis, Methylation, Cervical lesion

## Abstract

**Background:**

The dynamic methylation of human papillomavirus (HPV) 16 DNA is thought to be associated with the progression of cervical lesions. Previous studies that did not consider the physical status of HPV 16 may have incorrectly mapped HPV 16 methylomes. In order to identify reliable biomarkers for squamous cervical cancer (SCC), we comprehensively evaluated the methylation of HPV 16 depending on the integration incidence of each sample.

**Methods:**

Based on the integration status of 115 HPV 16-infected patients (50 SCC, 30 high-grade squamous intraepithelial lesion [HSIL], and 35 low-grade squamous intraepithelial lesion [LSIL]) and HPV 16-infected Caski cell lines by PCR detection of integrated papillomavirus sequences, we designed a series of primers that would not be influenced by breakpoints for a high-resolution melting (HRM) PCR method to detect the genome methylation.

**Results:**

A few regions with recurrent interruptions were identified in E1, E2/E4, L1, and L2 despite scattering of breakpoints throughout all eight genes of HPV 16. Frequent integration sites often occurred concomitantly with methylated CpG sites. The HRM PCR method showed 100% agreement with pyrosequencing when 3% was set as the cutoff value. A panel of CpG sites such as nt5606, nt5609, nt5615, and nt5378 can be combined in reweighing calculations to distinguish SCC from HSIL and LSIL patients which have high sensitivity and specificity (88% and 92.31%, respectively).

**Conclusions:**

Our research shows that combination of CpG sites nt5606, nt5609, nt5615, and nt5378 can be used as potential diagnosis biomarkers for SCC, and the HRM PCR method is suitable for clinical methylation analysis.

**Electronic supplementary material:**

The online version of this article (10.1186/s13148-018-0445-8) contains supplementary material, which is available to authorized users.

## Background

High-risk human papillomavirus (HPV) 16 is known to be closely associated with cervical squamous cell carcinoma (SCC) worldwide. Persistent HPV infections are considered to drive progression of cervical neoplasia to invasive cervical cancer [1]. Widespread screening of women with the Papanicolaou test has reduced the incidence and mortality due to cervical cancer in countries where it has been systematically implemented. However, some cases are still missed, and otherwise, repeat colposcopy is invasive and difficult to bear for some women. In 2015, the American Society for Colposcopy and Cervical Pathology (ASCCP) and the Society of Gynecologic Oncologists (SGO) convened to provide interim guidance for primary high-risk HPV screening in which high-risk HPV test can be considered alone as alternative to current cervical cancer screening methods [2]. As HPV testing cannot distinguish a persistent infection from a transient infection, it is difficult to predict the risk of cervical cancer from this test alone. Over the past few years, introduction of the HPV vaccine has provided evidence of reduced HPV 16/18 infections in countries where there has been high coverage and those who still have no chance to receive HPV vaccine such as Chinese women are increasingly concerned about high-risk HPV infection [3]. A more accurate and sensitive method than HR-HPV test is demanding that can predict risk of developing into cervical cancer.

DNA methylation is thought to occur early in malignant transformation, and methylation has recently been investigated as a more stable biomarker [4]. HPV 16 has a high GC content with part of non-traditional CpG islands. The LCR and L1 genes of HPV 16 have been the focus of most attention [5, 6]. Hypermethylated CpG sites in E2 binding sites are thought to repress E2 expression, resulting in deregulation of the E6/E7 oncogene which leads to carcinogenesis [7]. Alternatively, methylated CpG sites in the L1 gene are assumed to serve as a defense mechanism against the host’s immune system, helping development of persistent infection [8]. Like many other double-stranded circular DNA viruses, HPV often integrates into the human genome, which has previously been suggested to contribute to disease progression [9]. Kalantari et al.’s laboratory research firstly demonstrated that integration of the viral genome into the host chromosome events leads to an alteration in methylation patterns on the viral genome that is dependent upon the type of integration event in selected cell lines [10]. And increased methylation of human papillomavirus type 16 DNA was proved to correlate with viral integration in Bryant et al.’s few vulvar intraepithelial neoplasia samples and all this addressed the need to combine the two factors in any related research [11]. Those investigations either treat both integration and methylation as independent factor in their research or purely detect the methylation of the whole genome while disregarding any influence of the integration status that may illustrate a disguised map of methylation status of their samples [12, 13]. Furthermore, due to the different sample types and methodology used by these studies, there is no uniformity of the results. The conclusions of some studies are even conflicting [12–15].

Recent high-throughput sequencing of HPV 16 DNA in patients of different cervical lesions, combined with cost-effective target selection system technology, has revealed a greater number of tandem repetitive integration events, altered chromatin, copy number variation, and new integration hot spots, making it more complex to calculate the methylation level of HPV 16 [16]. Integration has long been thought to be associated with persistent infection, and frequent interruptions exist within the human genome. However, evaluating integration is not suitable for clinical application due to the irregular appearance in clinical samples, and the underlying pathogenic mechanism remains unclear [17, 18]. Furthermore, the interplay mechanism between integration and methylation and the extent to which integration can influence methylation detection are rarely reviewed especially in clinical sample.

Further knowledge regarding the methylation of HPV 16 considering integration incidence may assist our understanding about pathogenesis modulated by epigenetic mechanisms. In this study, we established a detection of integrated papillomavirus sequence (DIPS) PCR method to investigate the integration status of SCC, high-grade squamous intraepithelial lesion (HSIL), and low-grade squamous intraepithelial lesion (LSIL) samples. Based on the integration status, we designed a series of primers that would not be influenced by breakpoints in order to establish a high-resolution melting (HRM) method to detect the genome methylation. All results were confirmed by pyrosequencing.

## Methods

### Origin of samples

A total of 480 patients with HSIL or LSIL for whom Pap smear samples were collected (ThinPrep system, Hologic, Germany) at the Obstetrics and Gynecology Hospital affiliated with Fudan University between October 2015 and April 2016 were enrolled in the study. Pathological diagnosis was made based on colposcopy-guided punch biopsy, and cytological diagnosis was made based on the Bethesda system (TBS; revised in 2001). Patients whose cytological diagnosis was consistent with their pathological diagnosis were included in this study. Eighty SCC tissue samples were collected from the tissue bank of the hospital. All samples were stored at − 80 °C until use. Caski (ATCC® CRL-1550™) and HeLa (ATCC® CCL-2™) cell lines were purchased from ATCC. DNA was extracted from the cell lines and clinical samples using Sangon magnetic-bead purification kits (Sangon Biotech, China) which can obtain intact DNA. HPV typing was performed by PCR using universal primers MY09/MY11 and sequence confirmation. A total of 115 specimens were found to be positive for HPV 16 DNA (50 cases of SCC, 30 cases of high-grade squamous intraepithelial lesion [HSIL], and 35 cases of low-grade squamous intraepithelial lesion [LSIL]). The DNA of each sample was divided equally into two EP tubes, one for DIPS PCR and the other for HRM PCR. The study was approved by the institutional ethics committee. All patients signed an informed consent form.

### Detection of integrated papillomavirus sequence (DIPS) PCR analysis of HPV 16 integration status

#### Primers and double-strand adapter sequences

Fifteen pairs of primers were designed using Primer Premier 6 software based on previously reported interrupted sites in the HPV 16 genome (see Table 1). The sequences of the double-strand adapter and procedure were followed as described by Luft et al. [19] with some modifications. These modifications included only using Sau3AI, and we added the Titanium Taq DNA Polymerase and PrimeSTAR DNA polymerase (Takara, Japan) into the reaction mixture to amplify genomic DNA templates of all sizes.

**Table 1:** Fifteen pairs of primers were designed using Primer Premier 6 software based on previously reported interrupted sites in the HPV 16 genome

Primer	Sequence (5′–3′)	Size (bp)
E2BSs	F-ccgaccccttatattatggaatctt	
E2BSsa	B-cagatgtctttgcttttcttcaggaca	1461
E1-1	F-atatcagatgacgagaacgaaaatga	
E1-1a	B-gatacaggtgaagatttggtagattttatagta	2489
E1-2	F-gaaacaccatgtagtcagtatagtg	
E1-2a	B-agtgggggtggtagcagtcagtatagtagtggaag	2126
E1-3	F-gaattatcacagatggtacaatg	
E1-3a	B-cgataatgacatagtagacgatagtga	1521
E2-1	F-gtaaaacataaaagtgcaattg	
E2-1a	B-acttacatatgatagtgaatgtcaacg	763
E2-2	F-agttgttgcacagagactccgt	
E2-2a	B-agtgctccaatcctcactgcag	975
E2-3	F-ctttggtgcccaaggcgacg	
E2-3a	B-tatgggtcgcggcggagtggttggcc	2553
E2-4	F-tgatgtgtatgtagacacagacaaaagcagcggacgta	
E2-4a	B-ataggcagacacacaaaagcaca	450
L2-1	F-tagaattacaaactataacacc	
L2-1a	B-tctacatatacaaccacttcccatgc	779
L2-2	F-agtacgcctagaggttaatgctg	
L2-2a	B-ctggcctatgtaaagcaactata	121
L2-3	F-ataatgtcaggtggac	
L2-3a	B-catgttttataaagttgggtggc	821
L1-1	F-tccatagatatacattctct	
L1-1a	B-ctgtgtcatccaatttatttaata	791
L1-2	F-caagcaattgcctgggatg	
L1-2a	B-ctataagtatcttctagtgtgc	740
L1-3	F-gcaaaggatccccatgtaacaatg	
L1-3a	B-caggtgattgtccaccattagagttaat	804
E6-E7	F-cttatattatggaatctttgc	
E6-E7a	B-gtccagatgtctttgcttttc	1522

Primer sets for DIPS-PCR of HPV16 integration analysis. Primers without subscript are used for the first linear PCR and with a subscript are used for the second exponential PCR

#### Linear and exponential PCR

The first phase of linear touchdown PCR was performed as follows: denaturation at 94 °C for 5 min, followed by 40 cycles consisting of denaturation at 94 °C for 30 s, annealing for 45 s from 62 to 50 °C (decrease of 0.2 °C per cycle), and extension at 72 °C for 45 s, and final extension at 72 °C for 10 min. The second phase of exponential PCR was carried out using the same conditions described above, except 4 μL of adapter-specific AP1 primers and 4 μL of virus-specific AP2 primers were added.

#### Sanger sequencing of the PCR amplicons

The PCR amplicons were electrophoresed on 1.5% agarose gels stained with 4S green (Sangon, China) and read by transillumination. Caski DNA was used as the control. Any products that were not in expected bands were excised from the gel, extracted using the Tiangen Gel Extraction Kit (Tiangen, China), and sent for direct Sanger sequencing (Invitrogen, Thermo Scientific, America). A few amplicons were subjected to TA cloning when direct sequencing was difficult due to a low concentration. All sequencing data were collected to analyze the integration status of each specimen, achieved using DNAstar software and the NCBI BLAST tool.

### High-resolution melting PCR analysis of HPV 16 genome methylation

#### Standardization of high-resolution melting PCR

The HRM primers were designed with Methyl Primer software to screen for methylation of a total of 61 CpG sites in the HPV genome, based on the results of the integration experiments, in order to avoid breakpoints in each sample (see Table 2). None of the targeted amplicons exceeded 200 bp due to the limitations of this method. Part of the unmethylated and methylated LCR, L1, L2, E1, E2/E4, and E6/E7 genes (see Table 2), including their own CpG sites, were synthesized by Sangon Company according to the HPV 16 genome downloaded from NCBI (Genbank JQ004098.1) and cloned into plasmids. Six pairs of the constructed plasmids were mixed together in a 0, 10, 25, 50, 75, and 100% methylated to unmethylated ratio to generate standard HRM curves for quantitative analysis. The DNA concentration of all amplified template sequences was combined to analyze the accuracy of the HRM method. DNA concentration was confirmed using a Nanodrop ND-2000 spectrophotometer (Thermo Scientific, America).

**Table 2:** The HRM primers were designed with Methyl Primer software to screen for methylation of a total of 61 CpG sites in the HPV genome, based on the results of the integration experiments, in order to avoid breakpoints in each sample

Primer	Sequence (5′–3′)	CpG sites	Size (bp)
E2BSs	F-AATTTATGTATAAAATTAAG	nt31, nt37, nt43,	106
	R-CCTAAAACATTACAATTCTCTT	nt52, nt58	
E2	F-TTGAAATTATTAGGTAGTATTTGGTT	nt3412, nt3415, nt3417, nt3433, nt3461, nt3462	
	R-TAATYGTCTATATTTCTTYGATG		90
L1-1	F-GTTTTTTGTAGATTTAGATTAGTTTTTTT	nt7034, nt7091, nt7136, nt7145	187
	R-AAACAACACTAATTCAACATACATACAA		
L1-2	F-TGTTAGAATTATATGGCGATAGTT	nt6367, nt6389, nt6457	124
	R-CCTTTAATATATAAATCGTCTAATACATT		
L1-3	F-TAATTTAGATATATAGCGGTTGGT	nt5927, nt5963	81
	R-CTAATACCCACACCTAATAACTAA		
L1-4	F-GAGTATAGGGTTATAATAATGGT	nt6581, nt6650	109
	R-AAATAAATATAACAACACATAATAACAT		
L1-5	F-TTAAGGAGTATTTACGATATGGG	nt6731, nt6796	199
	R-ACAATTACCTAAATTACAAACCTATA		
L1-6	F-TTTGATATATTTATTAATATAATTGATTA	nt5600, nt5606, nt5609, nt5615	142
	R-CYTTTACYTCYTTTTCYTAACATATA		
L1-7	F-TGTAAGTACGGATGAATATGTT	nt5709, nt5726	81
	R-AAATATCCAACTACAAATAATCTAAA		
L2-1	F-AATAAATTGTTATTATTTAATAATGCGAT	nt4240, nt4249, nt4261, nt4270, nt4277	120
	R-ATAATATCAAATAAACATATACCTACCTA		
L2-2	F-TTTGATATTATATTTAAGGTTGAAGGT	nt4427, nt4437, nt4441	125
	R-CCAATACGTCCGCCTATAC		
L2-3	F-ATAGTAATTAGTAGTATATTTATATTAGG	nt4894, nt4906, nt4924	90
	R-TACAACTTTAACCTATTATATTATACG		
L2-4	F-TTAGGCGTATTGGTATTAGGT	nt5128, nt5173, nt5179	101
	R-ATCATAATAATAATATACCTTAACACCT		
L2-5	F-TTTTATAATTTYGGTAGGATTTGTA	nt5378	150
	R-CTTAATCAATTATATTAATAAATATATCAA		
E6	F-TTGTTAATTAGGTGTATTAATTGTTAA	nt494, nt502, nt506, nt539	179
	R-TATATCTCCATACATAATTACAACTAA		
E7	F-GATAAGTAGAATCGGATAGAGTT	nt701, nt752, nt757, nt765, nt780, nt789	137
	R-CCTAATATACCCATTAACAAATCTT		
Clone-LCR	F-CCTACTAATTGTGTTGTGGT		689
	R-CTCCTGTGGGTCCTGAA		
Clone-E6	F-GGAACAACATTAGAACAGCA		231
	R-GCAATGTAGGTGTATCTCCA		
Clone-E7	F-GCAACCAGAGACAACTGA		231
	R-GGGCACACAATTCCTAGT		
Clone-E2	F-AGCAACGAAGTATCCTCTC		187
	R-TGTCCACTGAGTCTCTGT		
Clone-L2	F-CCTCTGCGTTTAGGTGTT		1504
	R-GAAGGAGCTTGGTCAGTTAT		
Clone-L1	F-TTCCAGGGTCTCCACAATA		1693
	R-GCACATACAAGCACATACAA		

Primers for high-resolution melting PCR for determination of human papillomavirus 16 methylation and construction of standard curves

#### High-resolution melting PCR analysis of clinical samples and cell lines

Bisulfite conversion of DNA (0.8 μg) of the 115 clinical samples and the Caski and HeLa cell lines was performed using the EpiTect Bisulfite Kit (Qiagen, Germany) according to the manufacturer’s protocol. All unmethylated cytosines in the genome, excluding 5-methyl-cytosines, were converted to uracil and ultimately to thymine during Taq polymerase amplification. The HeLa cell DNA was used as a negative control.

PCR amplification of the converted DNA was carried out in a 25-μL reaction volume containing 18 μL of 2× EpiTect HRM PCR Master Mix (Qiagen, Germany), 1 μL of each primer (10 pmol/μL), 2 μL RNase-free water, and 3 μL of template DNA. PCR amplification and HRM were performed in a Light Cycler 480 (Roche) with the following parameters: denaturation at 94 °C for 5 min; 45 cycles of touchdown PCR consisting of denaturation at 94 °C for 30 s, annealing for 30 s from 65 to 50 °C (decrease of 0.2 °C per cycle), and extension at 72 °C for 30 s; and final extension at 72 °C for 10 min. The HRM procedure was as follows: 95 °C for 1 min, 40 °C for 1 min, 65 °C for 1 s, and then continuous acquisition until 95 °C in 0.02 °C increments. Samples were all run in duplicate in parallel with the standards. Genescanning software (Roche) was used to normalize the melt curves, and the temperature-shifted and distinct melting curves of each sample were generated. The results and efficiency of bisulfite conversion were confirmed by pyrosequencing.

### Statistical analysis

We used SPSS 21.0 software for all statistical analyses. Linear regression analysis was used to evaluate the association between the results obtained by HRM and the pyrosequencing method. To analyze differences in the levels of methylated CpG sites among different cervical lesions, the non-parametric Kruskal–Wallis and Mann–Whitney *U* tests were employed. The receiver operating characteristic (ROC) curves were used to calculate the optimal cutoff value and evaluate the negative predict value (NPV) and positive predict value (PPV) for each of the CpG sites. Multiple linear regression equation and cross-validation were used to reweigh each CpG site for clinical diagnostic. Statistical assays were all two-sided, and *P* < 0.05 was considered to be statistically significant.

## Results

### Analysis of HPV 16 integration in cell lines

**Fig. 1:**
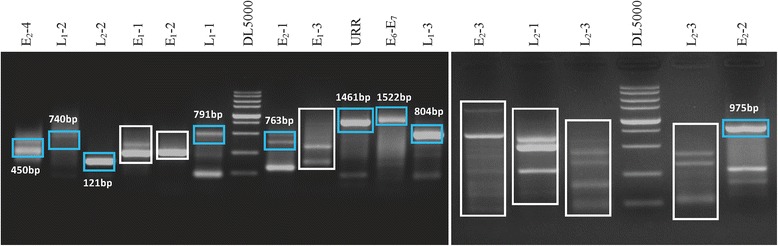
Confirmation of the primers designed for calculating integration in the Caski cell line. PCR products were electrophoresed on two 1.5% agarose gels that were run at the same time. The primer used is indicated above the band. DL 5000 was used as the reference marker (Takara, Japan), with bands from the top to the bottom representing 5000, 3000, 2000, 1000, 750, 500, 250, and 100 bp, respectively. DNA bands that were not the expected size are shown in a small box, and these fragments were sent for direct sequencing. There were some recurrent interruptions, and the different band sizes occurred in the same lane

Fifteen pairs of primers were used to identify integration in the Caski cell line using DIPS PCR. We successfully detected three types of integration in the Caski cell line, 11q14.1, 2q24.1, and Xq27.3, as previously reported by Meissner [20] (Fig. 1). No amplification was found in the HeLa cell line (data not shown).

### Identification of HPV 16 integration in the clinical samples

**Fig. 2:**
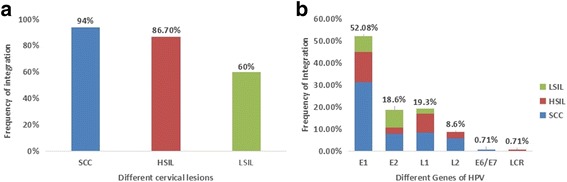
**a** Integration frequency of squamous cervical cancer (SCC), high-grade squamous intraepithelial lesion (HSIL), and low-grade squamous intraepithelial lesion (LSIL) (*P* < 0.001, *χ*^2^ = 16.605). **b** Frequency of integration events of different HPV genes in different groups. All interruptions occurred in these genes, including multiple integration events in the same gene of the same specimen (*P* < 0.001, *χ*^2^ = 185.83)

Overall, 81.7% of the 115 clinical samples demonstrated integration. The frequency of integration in SCC, HSIL, and LSIL patients was 94% (47/50), 86.7% (26/30), and 60% (21/35), respectively. There was a significant difference between the three groups (*P* < 0.05, Fig. 2a). Most of the integration events were located in previously reported hot spots in the human genome (see Table 3) [21]. However, there were no obvious preferential sites in the human genome among the three groups. Furthermore, disruption of HPV 16 was scattered throughout all genes from LCR to L1, with frequencies of 0.71% (LCR), 0.71% (E6/E7), 52% (E1), 18.6% (E2), 8.6% (L2), and 19.3% (L1) moving clockwise, as shown in Fig. 2b (*P* < 0.001, *χ*^2^ = 185.83). The relationship between HPV integration and clinicopathological characteristic of the three groups are see in Table 4. There was no correlation between integration in genes and the grade of cervical lesions. However, we noticed that there were more commonly interrupted regions of the HPV genome, including nt1800–nt2400 and nt2600–nt2900 in E1 (63/73), nt3200–nt3600 in E2/E4 (17/26), nt5300–nt5500 in L2 (19/27), and nt5600–nt5900 in L1 (8/12). In addition, several recurrent disruption sites were found, including nt2735 (*n* = 6), nt3467 (*n* = 5), nt1899 (*n* = 8), nt5365 (*n* = 4), and nt5619 (*n* = 4), some of which were very close to the CpG sites, and these will be a focus of future research (Fig. 4). Here, the recurrent interruption sites must contain more than three breakup events and more than five interruptions usually happened in the so-called commonly high interruption region. The promoter gene and oncogene E6/E7 showed little interference by interruptions (1/140 and 1/140, respectively).

**Table 3:** Most of the integration events were located in previously reported hot spots in the human genome

Gene	Locus	Frequency	Virus breakpoint sites
*MYC*	8q24	6	nt3467, nt3392, nt1886, nt1960, nt2210
*SLC7A1*	1p32.3	2	nt3467, nt3371
*MYF5*	12q21	1	nt5619
*SLITRK5*	3q26.1	2	nt3867, nt1974
*RAB39A*	10q26.11	2	nt5619, nt5311
*CD53*	1p13.3	2	nt5834, nt2663
*TMEM219*	16p11.2	2	nt5320, nt5365
*BRCA1*	17q21	3	nt3467, nt1869, nt2331
*ZFP91*	19q12	5	nt5812, nt5789, nt1890, nt2759, nt2860
*LOC642587*	1q32.2	1	nt5365
*SLIT3*	5q35.1	1	nt3272
*ZNF595*	4p16.3	3	nt5623, nt5521, nt2601
*CD34*	1q32.2	2	nt5619, nt2775
*CDK14*	7q21.13	2	nt5796, nt1903
*RAB9B*	Xq22.2	1	nt5353
*STK24*	13q32	2	nt2254, nt2684
*SH3BP2*	4p16	1	nt5296
*SLITRK2*	Xq27.3	4	nt5794, nt5321, nt5561, nt2117
*TNFAIP2*	14q32.3	2	nt2735, nt2115
*ZNF519*	18p11	2	nt5826, nt2260
*TNFSF15*	9q32	1	nt5207
*SLC4A3*	2q35	2	nt2789, nt2334
*MYO1H*	12q24	1	nt5897
*SPANXN1*	Xq27.3	2	nt2677, nt2892
*MRPL17*	11p15.4	2	nt1908, nt2741
*OR2AG1*	11p15.4	2	nt5783, nt2332
*FANCC*	9q22	2	nt3467, nt2467
*SLC1A3*	5p13.2	1	nt2887
*LRP1B*	2q21	2	nt5811, nt1899
*SOX6*	11p15.2	2	nt2434, nt2561
*LOC401097*	3q25	1	nt3592
*SLC13A1*	7q31	1	nt1899
*MYBPC1*	12q23	1	nt2770
*SLC38A4*	12q13	2	nt1889, nt2654
*CYP7A1*	8q12	2	nt2735, nt1899
*ATPBD4*	15q14	2	nt2017, nt2229
*SOX2*	3q26	3	nt3226, nt2115, nt2095
*CADM2*	3p12	3	nt2735, nt1899, nt2833
*LOC728410*	10q26	2	nt3309, nt3596
*PRKX*	Xp22	3	nt5964, nt5790
*GAP43*	3q13	1	nt2735
*TFPI*	2q32	2	nt3584, nt2607
*PPP2R3A*	3q22	2	nt2774, nt2601
*MCTP1*	5q15	1	nt3206
*HLF*	17q22	2	nt5934, nt5821
*CLTCL1*	22q11	1	nt3201
*LPPR4*	1p21	2	nt5797, nt2735
*SLC5A3*	21q22	1	nt1899
*ZNF165*	6p22	1	nt3298
*STK24*	13q32.2	1	nt2392
*C5orf39*	5p12	1	nt1899
*PCDH17*	13q21	1	nt3577
*STK11*	19p13.3	1	nt1923
*TMEM26*	10q21	1	nt3318
*HLA-E*	6p22	1	nt5867
*FAM82A1*	2p22.2	1	nt2397
CD44	11p13	2	nt3215
*FAM131C*	1p36.13	1	nt6854
*SLBP*	4p16.3	1	nt2662
*SOX17*	8q12.1	1	nt2735
*PRKCA*	17q24.2	1	nt3596
*PAH*	12q23.2	1	nt7458

Integration gene and frequency of the human genome and breakpoints of HPV16 occurred in all samples

**Table 4:** The relationship between clinicopathological characteristic of three groups and HPV integrations

Variable	No. of patients	HPV integrations				*P* value
		Negative		Positive		
		No.	%	No.	%	
SCC patients						
Age						
< 40	6	0	0	6	100	0.99
40–50	32	2	6.25	30	93.8	
> 50	12	1	8.33	11	91.7	
Stage						
Stage I	13	0	0	13	100	0.558
Stage II+	37	3	8.1	34	91.9	
Metastasis						
Negative	42	2	4.76	40	95.2	0.414
Positive	8	1	12.5	7	87.5	
Number of pregnancies						
< 3	21	1	4.76	20	95.2	0.99
≥ 3	25	2	8	23	92	
Number of abortions						
< 2	22	3	13.6	19	86.3	0.079
≥ 2	28	0	0	28	100	
HSIL and LSIL patients						
HSIL	30	4	13.3	26	86.7	0.017
LSIL	35	14	40	0.6	60	
Age						
< 40	52	7	13.5	45	86.5	0.19
40–50	11	4	36.4	7	63.6	
> 50	2	0	0	2	100	

This table shows both the clinicopathological characteristic of SCC, HSIL, and LSIL and their frequency of HPV integrations. *P* < 0.05 is considered to be significantly different

### Standards for high-resolution melting PCR

All dilutions of plasmids were successfully amplified with the corresponding primers. Each of the curves generated from HRM PCR exhibited a single peak with a specific melting temperature. All methylated plasmids could be distinguished from dilutions containing 0–100% methylated plasmids. The quantitative standard curves for calculating the methylation of different CpG sites are shown in Additional file 1: Figure S1. Several linear analyses were used to assess the linearity of the methylation test, correlation coefficient, and detection limit (see Additional file 1: Figure S1 and Table 5). No amplification was detected for the HeLa cell DNA (data not shown). There was no non-specific amplification of standard plasmids, as confirmed by subsequent direct sequencing.

**Table 5:** The fluorescence peak height of a dilution of methylated template in the background of unmethylated template and their correlation coefficiency of different gene standards for methylation analysis of HRM-PCR

Genes	Standards						
	Fluorescence peak height						Correlation coefficient
	0%	1%	25%	50%	75%	100%	
ESBSs	69.20	51.00	42.50	33.60	21.90	5.80	0.963
L1-1	76.10	42.20	35.70	24.50	14.80	5.10	0.971
L1-2	66.20	46.70	38.20	24.00	12.30	5.80	0.983
L1-3	56.30	48.80	40.20	28.50	12.30	3.60	0.976
L1-4	77.80	47.60	32.80	25.40	47.20	7.20	0.919
L1-5	89.40	70.90	55.00	39.00	15.00	6.90	0.953
L1-6	66.90	49.30	32.10	25.00	12.30	2.50	0.989
L1-7	60.40	55.00	41.60	18.00	12.00	2.70	0.963
L2-1	70.30	51.90	45.00	32.50	19.30	6.10	0.967
L2-2	69.80	44.80	32.10	22.30	10.50	1.60	0.985
L2-3	65.00	35.40	23.00	21.10	13.20	2.10	0.984
L2-4	67.50	47.70	39.00	25.90	12.30	2.10	0.959
L2-5	70.30	41.50	30.00	21.30	15.00	2.70	0.978
E2	66.00	55.90	42.10	23.80	12.20	2.50	0.950
E6	68.90	47.20	35.70	30.10	24.50	7.90	0.987
E7	66.70	55.90	48.10	26.70	25.20	5.80	0.958

### Analysis of HPV 16 genome methylation

**Fig. 3:**
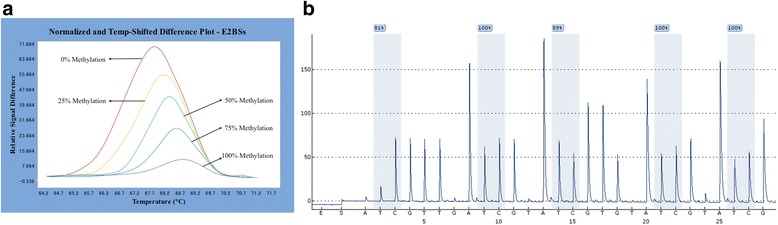
**a** Representative HRM standard curves of E2BSs. **b** The pyrosequencing results of 100% methylated standard plasmid of E2BSs

**Fig. 4:**
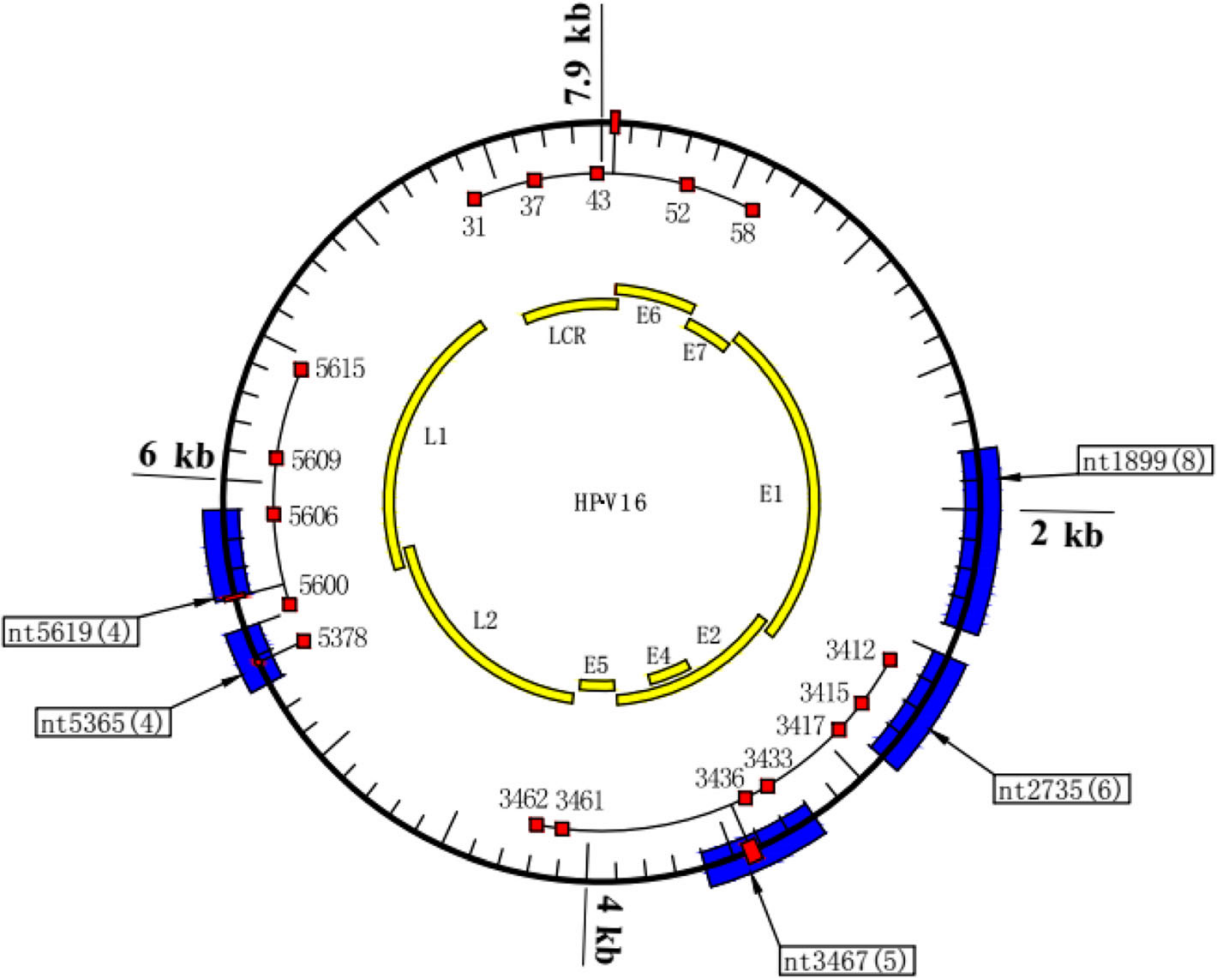
Recurrent breakpoints and methylated CpG islands near interruption sites are shown in the above map of the HPV 16 genome. The arrows indicate the location and the attached boxes state the number of integrations identified in this study. The red boxes represent the nearby methylated CpG sites. The frequently interrupted regions of E1, E2/E4, L1, and L2 are represented by the blue boxes

All 115 tissue samples and the Caski cell line were successfully amplified in parallel with five pairs of standard plasmids, among which 44 CpG sites showed no or lower methylation (Additional file 1: Figures S1 and S2). We were unable to calculate the methylation of CpG sites in the E1 gene from nt2655 to nt2754 due to its high degree of interruption using current method. In order to verify the accuracy of the HRM PCR method, all positive samples were also subjected to pyrosequencing to identify the methylated CpG sites (Additional file 1: Figure S2). When the cutoff values of pyrosequencing for detecting standard plasmids were set as 3%, the two methods showed 100% agreement (Additional file 1: Figures S1 and S2, Fig. 3). In addition, there were some differences in CpG site location using the HRM method, where different CpG sites in the same CpG island showed differences to the pyrosequencing method (Additional file 1: Figure S2). We found that several CpG sites including nt31, nt37, nt43, nt52, and nt58 were hypermethylated in SCC and HSIL compared to LSIL samples, all of which had a good PPV for distinguishing SCC and HSIL from LSIL (87%, 86.3%, 90.5%, 85.7%, 84.6% and 83.1%, respectively; *P* < 0.05) (Additional file 1: Figure S3). CpG sites nt3412, nt3417, nt3433, nt3436, nt3461, nt3462, nt5600, nt5609, nt5615, and nt5378 showed a high level of methylation in SCC, but a lower level of methylation in HSIL and LSIL which is consistent with Jacquin et al.’s result [7]. Based on the above results, we further reweighed CpG sites nt5606, nt5609, nt56015, and nt5378 and adjusted the multiple correlation coefficiency (multiple *R* = 0.84, *P* < 0.001) for the relatively small numbers of samples in equations which showed good sensitivity and specificity for distinguishing SCC (see Table 6, *Y* > 0.5) from HSIL and LSIL (0 < *Y* < 0.5) (88% and 92.31%, respectively; *P* < 0. 001). These panels of CpG sites are recommended for potential candidate biomarkers combined with other factors for diagnostic test of cervical cancer (Table 6). No defined methylation pattern was found in the three groups, which does not agree with a previous report [22]. Interestingly, we noticed that the high-frequency breakpoints were often accompanied by methylated CpG sites (Fig. 4). A series of CpG sites nt5600, nt5606, nt5609, and nt5615 resides in the commonly interrupted region nt5600–nt5900, and CpG sites nt3462, nt3461, nt3433, nt3417, nt3415, nt3412, and nt5378 are often imbedded in the interruption region nt5300–nt5500 which near the recurrent interrupted sites nt3467 [5] and nt5365 [4] respectively.

**Table 6:** Sensitivity and Specificity of the multiple linear regression equation using a panel of CpG sites

Patient numbers of SCC group	Patient numbers of predictive SCC group	Patient numbers of predictive HSIL and LSIL group	Total numbers of each defined group	Sensitivity% (*P* < 0.01)	Specificity% (*P* < 0.01)
Patient numbers of SCC group	44	6	60	88	
Patient numbers of HSIL and LSIL group	5	60	65		92.31

The multiple linear regression equation is as follows: *Y* = 0.006X1 + 0.15X2 + 0.29X3 + 0.93X4 − 0.008; multiple correlation coefficiency *R* = 0.84, adjusted *R*^2^ = 0.70, *P* < 0.01; X1, X2, X3, and X4 represent the value of CpG sites nt5606, nt5609, nt5615, and nt5378, respectively; when *Y* > 0.5, this is determined as positive, and when 0 < *Y* < 0.5, this is determined as negative

## Discussion

A large number of studies have evaluated HPV 16 methylation using traditional methods [23–26]. To the best of our knowledge, we are the first research group to evaluate HPV 16 methylation based on its integration status. The DIPS PCR approach we used is labor-intensive but costs less than next-generation sequencing (NGS) and fulfilled our requirements [27]. We found significantly integration difference among the three groups (*P* < 0.05), of which the higher integration frequency compared to previous study may be due to different DNA qualities, groups of histology, or analysis methods [28, 29]. As magnetic-bead purification kits are used in our research, it can provide us with intact high-quality DNA template which is vital to the whole experiment. Moreover, we added a mix of effective enzymes expert in producing amplicons of different sizes that cannot be found in any other similar assay [30].There were also several recurrent interruptions in the HPV 16 genome which seems to be a series of preferential interruption regions in specific genes. E1 was the gene most likely to be interrupted, and the integration usually appeared between nt1800–nt2400 and nt2600–2900. The E2/E4, L1, and L2 genes showed greater integration in nt3200–nt3600, nt5600–nt5900, and nt5300–nt5500, respectively, of which virus and cellular fusion usually occurred within the 3′-region of the HPV 16 gene. And those highly interrupted region also occurred in Liu et al.’s investigation who tried to enhance the understanding of the precise location of HPV16 integration sites using capture technology combined with next-generation sequencing [31]. In contrast, the E6/E7 oncogene and the promoter gene were rarely interrupted in our research which is lower compared to Liu et al.’s results. And there are also other interruption sites in Caski cell line except the three detected in our research revealed by NGS method [32]. This may partly ascribed to the sensitivity of the limited restriction enzyme methods we used.

Based on the integration status, we used an HRM PCR method to screen the methylation of integrated HPV 16 genome. This versatile approach can be used to analyze SNP, for human leukocyte antigen (HLA) matching and for species identification which has recently been described for methylation analysis [33–35]. In addition to HRM, several other methods have been used to analyze methylation status, each with their advantages and disadvantages. Methylation-specific PCR (MSP) is a traditional method that requires two pairs of primers to be designed for one target gene. This method is not very sensitive and cannot provide any quantitative information. MethyLight is a real-time method which is more complex and expensive [36]. In contrast, HRM PCR allows for the efficient screening of a sample to rapidly detect the methylation level and is more cost-effective than pyrosequencing which counts every CpG site and requires a specialized instrument [37]. In our research, the HRM method was able to distinguish methylated DNA at a level of 1% from non-methylated DNA (see Additional file 1: S1, S2 and Table 5). It is a reliable and sensitive method and is more convenient for clinical application when compared with pyrosequencing. All of the expected CpG sites were successfully detected using carefully designed primers. Some of the CpG sites in L1, L2, E2, and E2 binding sites showed different methylation levels among the three groups. The E2 binding sites were always hypermethylated in SCC and HSIL and hypomethylated in LSIL, whereas no methylation was detected in the E6/E7 gene in the current study. Epigenetic changes possibly contributed to deregulation of the E2 and E6/E7 gene due to the low frequency of interruption [38]. The non-methylated status of the E6/E7 gene may be partly due to the lack of integration detected by our method [39]. We could not calculate the methylation level of E1 due to the intensive breakpoints within or near the CpG sites; to date, no studies have reported methylation of the E1 gene and its interruption may be responsible for neoplasia progression during the virus life circle [40]. We also found limited interruption in E2, and the CpG sites surrounding the frequently interrupted sites were always hypermethylated in SCC. Methylation of E2, which is mainly responsible for E2 repression, showed no interruption effect compared to previous report [41]. We did not detect any methylation of the 3′-region of the L1-terminus in our sample in contrast with previous inconsistent methylation of the L1 terminus [42, 43]. Hypermethylation of CpG sites in the L1 and L2 genes may be associated with persistent infection by changing the capsid structure, which show no expression in the permissive stage. We further evaluated the most reliable CpG sites nt5606 (NPV = 86.3%, PPV = 90.6%, *P* < 0.001), nt5609 (NPV = 90.9%, PPV = 85.9%, *P* < 0.001), nt5615 (NPV = 86%, PPV = 89.2%, *P* < 0.001), and nt5378 (NPV = 92.8%, PPV = 89.1%, *P* < 0.001) in reweighing calculation with adjusted multiple correlation efficiency using cross-validation for stabilizing multivariable linear regression model and found that the combination of these biomarkers better help us in diagnostic test of SCC (see Table 6). We observed that each sample had its own unique methylation pattern, but were unable to identify any defined pattern that could be used to distinguish the three groups. We speculate that some of the DNA methylome of HPV 16 in human cells may undergo dynamic changes at the same or different stages of cervical disease [44]. In addition, we noticed that the recurrent integration regions were usually accompanied by methylated CpG sites. In their research, Kalantari et al. observed 3′-region integration of the internal copies of the HPV 16 genome in the concatemer, with hypermethylation of the LCR gene, evaluated using clonal W12 lines derived from the HPV 16-positive cervical intraepithelial neoplasia (CIN) [10]. This supports the hypothesis that inserted copies of the HPV 16 genome are finally silenced by epigenetic changes. Other viruses such as HBV (hepatitis B virus) show that methylation of the HBx gene is in accordance with the methylation status of the flanking human gene [45]. And also in their HPV research, they showed the DNA methylation status of the integrated HPV16 genome was affected by the methylation status of the human genome flanking integration breakpoints in three neck and head carcinoma cell lines [46].Thus, we suppose that some HPV integrants might be activated or inactivated through self-methylation induced by flanking methylation status of host genome when integrated into human genome, leading to tumorigenesis. Whether this is the case for HPV16-induced cervical cancer remains to be confirmed.

For the co-existence of integration and episoma in our samples, the clear relationship between integration and methylation could be further addressed by evaluating mRNA expression or by performing single molecule sequencing. Furthermore, these recommended panel biomarkers in our research need to be assured and optimized the reweighting calculation in the larger multicenter clinical samples. Also, more sensitive method should be used to exhaust all possible integration due to the limitation of DIPS PCR. This research is the first methylation study to consider the genomic state of the virus genome. Comprehensive mapping of the methylomes in HPV 16 based on their integration sites obtained reliable data and will help us to better understand the universal methylation level of HPV 16 or may be the interplay with integration. The research described here highlights the importance of considering the physical status of the virus of which increasingly insert into human genome with the severe disease transformation before methylation detection and promoting new discoveries.

## Conclusions

Our research shows that frequent integration sites often occurred concomitantly with methylated CpG sites and combination of CpG sites nt5606, nt5609, nt5615, and nt5378 can be used as potential diagnosis biomarkers for SCC, and the HRM PCR method is suitable for clinical methylation analysis.

## Additional file

Additional file 1: The quantitative standard curves for calculating the methylation of different CpG sites. (XLSX 51 kb)

## Abbreviations

DIPS-PCR: Detection of integrated papillomavirus sequence
HPV: Human papillomavirus
HSIL: High-grade squamous intraepithelial lesion
LSIL: Low-grade squamous intraepithelial lesion
SCC: Squamous cervical cancer
